# Application of Solid-State Nanopore in Protein Detection

**DOI:** 10.3390/ijms21082808

**Published:** 2020-04-17

**Authors:** Yuhan Luo, Linlin Wu, Jing Tu, Zuhong Lu

**Affiliations:** State Key Lab of Bioelectronics, School of Biological Science and Medical Engineering, Southeast University, Nanjing 210096, China; 213161526@seu.edu.cn (Y.L.); 220181825@seu.edu.cn (L.W.)

**Keywords:** solid-state nanopore, single molecule detection, protein sequencing, protein conformation

## Abstract

A protein is a kind of major biomacromolecule of life. Its sequence, structure, and content in organisms contains quite important information for normal or pathological physiological process. However, research of proteomics is facing certain obstacles. Only a few technologies are available for protein analysis, and their application is limited by chemical modification or the need for a large amount of sample. Solid-state nanopore overcomes some shortcomings of the existing technology, and has the ability to detect proteins at a single-molecule level, with its high sensitivity and robustness of device. Many works on detection of protein molecules and discriminating structure have been carried out in recent years. Single-molecule protein sequencing techniques based on solid-state nanopore are also been proposed and developed. Here, we categorize and describe these efforts and progress, as well as discuss their advantages and drawbacks.

## 1. Introduction

Proteins are an important component of human cells and tissues. All the important components and physiological activities of the body need the participation of proteins, which catalyze and control all the cell processes. Protein analysis provides key information of most biological processes, the phenotype of a cell, and disease [1]. Therefore, the proteome may reflect the physiological condition of the cell or living body better than genome, but proteomic data are more difficult to obtain [2]. The information contained in proteins includes its sequences and spatial structure. With the accumulation of many DNA (deoxyribonucleic acid) sequences, many researchers have realized that simply having a complete genomic sequence is not sufficient to elucidate biological functions, because structural features such as protein subtypes and post-translational modifications cannot be derived from DNA sequences [1].

The proteomics research is facing certain obstacles. There is no amplification method for protein [3], and protein expression levels cannot be indirectly predicted from transcription levels [4,5]. Commonly used methods to evaluate the secondary structure of proteins, e.g. X-ray crystallography, nuclear magnetic resonance (NMR), circular dichroism (CD), and infrared spectroscopy (IR), cannot obtain dynamic information of proteins in real time. Ultrasensitive fluorescence microscope [6] and atomic force microscope (AFM) [7] can detect protein interactions and structural information. However, these methods require chemical modification on detected molecules or complex large-scale instruments, which are often expensive and cumbersome, and may damage the native properties of the protein, while complex experimental procedures may introduce more uncertainty. Tunneling currents are a way for single molecule detection [8]. Two electrodes are separated by a gap of a few nanometers or less. When individual molecules pass through the nanoscopic gap, a change in the tunneling currents is measured. This technique has been applied in measurements of a variety of biomolecules [9,10,11]. Recognition tunneling using a functionalized pair of tunneling electrodes can be adapted to read small drug molecules, metabolites, amino acids, and small peptides [10,12]. Conductive properties of graphene make it an ideal material for making nanogaps for sequencing [13,14]. As another single molecule biosensor platform, nanopore sensors monitor the analytes in real time through the changes of ionic currents, without chemical modification or labeling. The principle and detection method of nanopore technology is simple and easy to implement. Thus, nanopore sensors show outstanding capabilities in protein sensing due to their advantages of label-free and high-throughput operation.

Nanopore technology originated from the invention of the Coulter counter and the recording technology of single channel current. In 1976, Neher and Sakamann applied the tip of glass micropipettes on to the frog muscle cells to detect membrane potentials, and recorded single-channel ionic currents activated by acetylcholine for the first time [15]. The glass micropipettes were only 3-5 µm in diameter, thus isolating electrically a small patch of membrane. Sigworth et al. applied suction to the inside of the micropipette yielding a 10Gohm gigaseal, and reduced the noise during recording [16]. Their work produced patch clamp technology and promoted the practical application of nanopore sequencing technology [17]. The basic principle of nanopores is that, in a cavity filled with electrolyte, an insulating barrier membrane with nanoscale pore divides the cavity into two small chambers. When a voltage is applied to the electrolyte, individual molecules can pass through the pores and partially block the flow of ions through the nanopore. The ionic current level is temporarily reduced and recovers to its original level after the translocation. Some factors such as the size and surface characteristics of nanopores, voltage, and solution can affect the state of ionic current. The dwell time, amplitude of the current, and capture rate contain useful information about the molecule’s structure and dynamic motion.

Basically, there are two kinds of nanopores: biological nanopore and solid-state nanopore. In some cases, both are combined to complete specific functions [18]. In 1996, Kasianowicz et al. used *S. aureus* α-hemolysin inserted into a lipid bilayer to form a protein nanopore with its pore size wide enough to accommodate a single strand of DNA (stem diameter ≈ 2.6 nm, limiting aperture size ≈1.5 nm). This was the first nanopore device that demonstrated its ability to detect single-stranded nucleic acid polymer [19]. Hereafter, other biological nanopores such as MspA [20,21], phi29 motor protein nanopore [22], and ClyA [23] enriched the research of biological nanopore technology. Biological nanopores with characterized structures have shown their high sensitivity and resolution. However, biological nanopores are sensitive to buffer concentration, pH value, and other external conditions [22,23]. In contrast to biological nanopores, nanopores prepared by solid materials can be designed according to the size, structure, and surface properties of the detected molecules. Solid-state nanopores with adjustable pore size and robustness broaden the ranges of target biomolecules, device structures, and preparation materials and are suitable for integration with other platforms [21,22,23,24,25,26,27].

Li et al. reported DNA sensing using solid-state nanopores for the first time, with a 5-nm diameter pore [25]. A systematical overview of solid-state nanopores is summarized in reviews by Lee et al. [28] and Gonzalo et al. [29]. Solid-state nanopores can be fabricated by focused ion beam (FIB) [25], electron-beam drilling (EBD) [26], controlled dielectric breakdown (CDB) [27,30], and so on. Silicon nitride, SiO_2_, and graphene are frequently used materials. For preventing non-specific interactions or advancing functionality, solid-state nanopores can be modified or coated with various materials. Typical organic materials include polyethylene glycol (PEG) [31], fluid lipid coatings [32], and 3-aminopropyltriethoxysilane (APTES) for salinization [33,34]. Inorganic materials such as Al_2_O_3_ [35], SiO_2_ [36], and HfO_2_ [37] can be deposited by atomic layer deposition (ALD) and chemical vapor deposition (CVD), for better signal-to-noise ratio. There are reviews focused on these efforts to enhance the performance and sensitivity of the solid-state nanopore devices as a biomolecule sensor [38,39].

To date, in addition to promising applications in nucleic acids detection [36,40,41,42,43,44,45], solid-state nanopores have made great progress in molecular interaction [46,47,48], detecting protein structures or their aggregation states [49,50,51,52], and virus identification [53]. However, nanopore signals of proteins are harder to resolve due to diversity of amino acids and inhomogeneous charge, as well as fast translocation [54]. Herein, we mainly focus on the field of solid-state nanopore-based protein characterization, including the effect of protein charge and pH on translocation, interaction of proteins with other molecules, discrimination of protein structure, and conformation. Recent efforts and progress of protein sequencing based on solid-state nanopores is also discussed.

## 2. Detection of Proteins and Interactions with other Molecules

In 2006, Han et al. first reported translocation of a single bovine serum albumin (BSA) protein molecule across a 20-nm-thick silicon nitride membrane with a 50-nm diameter pore [55], proving its potential to detect proteins as a Coulter counter. Afterwards, many studies on protein at single-molecule level based on solid-state nanopore have been reported [50,54,56,57]. Each protein has a different amino acid sequence, three-dimensional structure, and charge profiles. When passing through a nanopore, this information is reflected in detected current signal. Various properties of proteins have been studied in the nanopore field based on this principle.

### 2.1. Effect of pH Regulation on Protein Translocation

**Figure 1:**
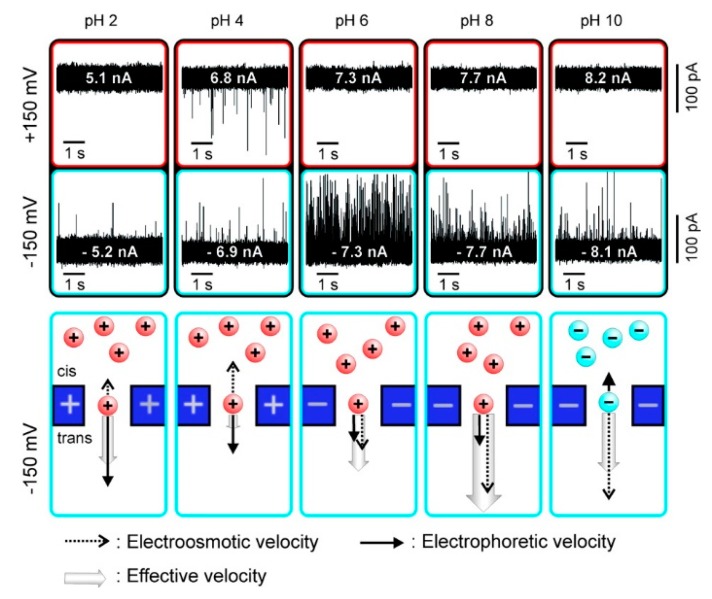
Current traces of avidin translocation through a silicon nitride nanopore and charge of protein and pore under different pH values. Adapted with permission from Nano Lett. 2010, 10, 6, 2162–2167. Copyright 2010 American Chemical Society.

As a kind of ampholytes, protein carries no net electrical charge at a certain pH, which is called the isoelectric point (pI). The net surface charge is affected by pH value so that the movement in an electric field may be changed. Firnkes et al. studied the factors affecting the transport direction of proteins in nanopores [58]. In addition to the electrophoretic force, they found that electroosmosis might have an effect that exceeds the electrophoretic force because the change of pH not only affects the charge of protein, but also the surface charge of nanopores. The direction and speed of proteins through nanopores in an electric field is governed by both electrophoretic and electroosmosis forces (Figure 1). When electrophoretic and electroosmosis forces offset each other, diffusion becomes the dominant contributor [58]. Likewise, Saharia et al. changed the net charge of human serum transferrin protein (hSTf), and they observed translocation events under both positive and negative voltage polarities at pH 4 (Table 1) [59]. They attributed the phenomenon to diffusion of the protein. Steinbock et al. used BSA to prove that the net charge of protein and its translocation can be affected by shifting pH higher or lower than the protein’s pI [51]. These works further proved the viewpoint above put forward by Firnkes et al. [58].

This principle helps to control the mode and speed of translocation. Nir et al. slowed down the translocation speed of ubiquitin by adjusting the pH value of the buffer near the pI, thereby realizing sensing and characterization of small globular proteins using solid-state nanopore [51]. At pH 7.2, most events could not be detected due to their extremely short dwell time. However, lowering the pH to 7.0 resulted in a significant increase in dwell time, making these events within a reliable measurement bandwidth [60]. Jubery et al. came up with an idea that electroosmosis and electrophoresis force could be utilized to separate nanoparticles [61]. They used high-density lipoprotein (HDL) and low-density lipoprotein (LDL) as the sample nanoparticles and designed a nanopore-based platform. Numerical simulations proved the feasibility of this idea. In other words, electroosmosis effect sometimes needs to be accounted for to help clarify the protein’s translocation.

### 2.2. Detection of DNA-Protein Complex

**Figure 2:**
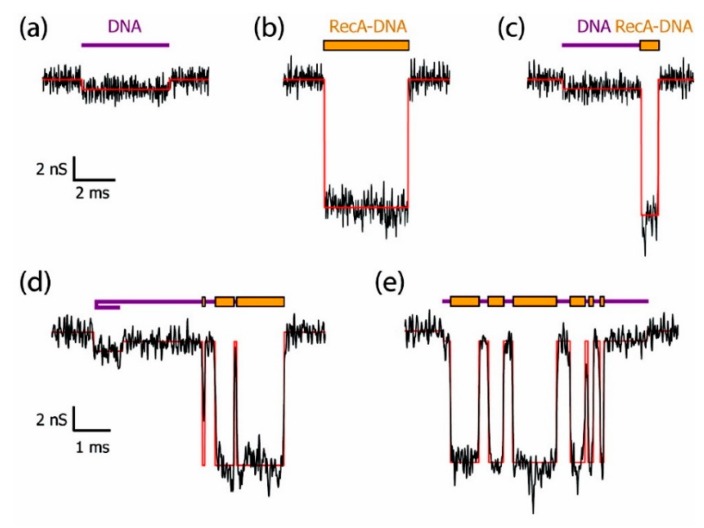
Example of current traces of differently RecA-coated DNA using a 30 nm diameter pore fabricated in 20 nm thick SiN membrane: (**a**) bare DNA; (**b**) fully RecA-coated DNA; (**c**) partially RecA-coated DNA; and (**d**–**e**) more complex events. Reprinted with permission from Nano Lett. 2010, 10, 1, 324–328. Copyright 2009 American Chemical Society.

As an important macromolecule in organisms, the interactions between proteins and proteins or other molecules are involved in many biological processes, and the structural and functional changes caused by these interactions have always been an important research area. Hall et al. successfully discriminated bare double-strand DNA (dsDNA) and RecA-dsDNA complex using a solid-state nanopore combined with an optical tweezer. The RecA proteins introduced negative charge, leading to 2–4 times larger electrophoretic force than the bare dsDNA so that they could be easily distinguished under salt conditions ranging from 100 mM to 1 M KCl [62]. Kowalczyk et al. locally detected proteins along DNA using silicon nitride nanopore of 30 nm diameter and 20 nm thick [63]. They coated DNA molecules with RecA proteins to form bare DNA, fully RecA-coated DNA, partially RecA-coated DNA, generating distinguishable current-blockade signatures (Figure 2). By analyzing voltage-dependence of translocation time, they found that the best resolution was observed in the 10–15 mV range where they obtained a spatial resolution of about 8 nm corresponding to 5 RecA proteins binding to 15 base pairs of DNA, which demonstrated high resolution of solid-state nanopores sensing.

**Figure 3:**
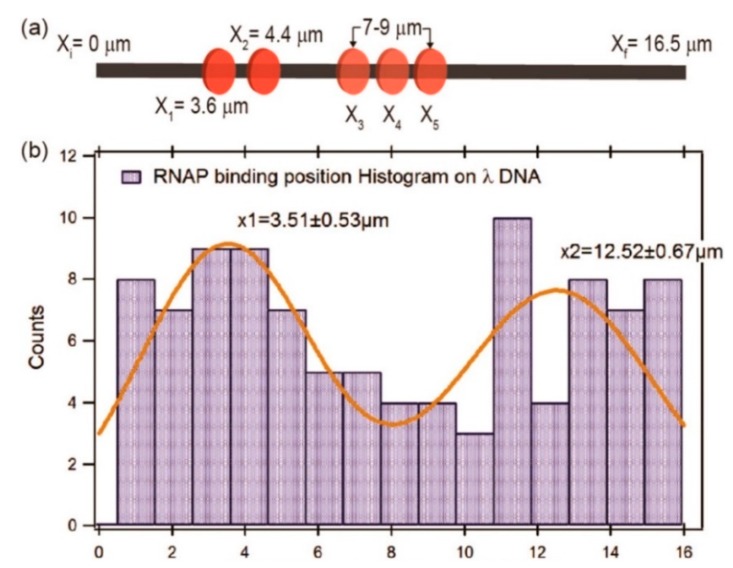
(**a**) Sketch map of ideal RNAP binding locations; and (**b**) histogram and Gaussians fitting of measured RNAP biding locations. Adapted with permission from ACS Sens. 2019, 4, 1, 100–109. Copyright 2018 American Chemical Society.

Raillon et al. first reported detection of a single *E. coli* RNA polymerase (RNAP)–DNA transcription complex and single *E. coli* RNAP using solid-state nanopore sensing [64]. They observed transcription complex’s translocation events. Interestingly, translocation events of the β or β′ subunit of RNAP were also observed at high voltage, possibly because high voltage separated them from the core enzyme. This work demonstrated the effect of voltage on protein structure. Recently, Kaur et al. studied the binding sites of RNAP on long λ DNA using a silicon nitride nanopore [65]. There are five biding sites on a λ DNA, two strong binding sites at 38,003 (3.55 μm) and 35,602 bp (4.40 μm) close to the 3′ end, and three weaker binding sites between 7 and 9 μm (at about 27,649, 25,620, and 23,619 bp). When RNAP crosses the pore, there would be a subevent. By analyzing the dwell time and amplitudes of current blockage signals and their subevents, as well as subevent starting times, they estimated the binding efficiency and locations of RNAPs on a λ DNA. It was found that RNAP had a high binding tendency at about 3.51 ± 0.53 μm, most likely corresponding to the two strong promoter regions of 3.48 and 4.43 μm on λ DNA, respectively (Figure 3). The peak at about x = 12.52 ± 0.67 μm was possibly because the λ DNA entered the nanopore in opposite orientation. However, due to the complex λ DNA folding structure and low time resolution, it is difficult to completely distinguish the total five binding sites on λ DNA. DNA–protein interactions have also been used to estimate protein quantitative information. Using DNA as a carrier, two systems were designed to detect nanomolar proteins [66]. DNA was modified with biotin or digoxigenin to detect streptavidin or anti-digoxigenin concentrations, respectively. When the protein is bounded to a DNA carrier, a typical secondary current drop occurs. By measuring the concentration dependence of the proportion of the corresponding signal, the protein concentration can be quantitatively estimated.

### 2.3. Detection of Antigen-Antibody Interactions

On the other hand, solid-state nanopore-based drug screening using antigen–antibody interactions is a promising research area. In 2006, a resistance pulse sensor based on a submicrometer pore was used to detect the ability of antibodies to immunoprecipitate virus and determine the number of antibodies bound to single virus particles [67]. However, it will be a challenge to detect small number of antibodies or other molecules. Freedman et al. detected the binding of HIV antigen, envelope glycoprotein gp120v and anti-gp120 antibody [68]. They observed a bimodal signal corresponding to the antibody monomers and dimers when characterizing antibody. Gp120 and its antibodies had different binding states. In addition to the monovalent binding that occupied the major part, there were also populations with larger block, which is likely due to antibody dimers with both monovalent and multivalent binding of gp120. They also calculated the excluded volume of the gp120–antibody complex, which was less than the sum of volume of the two molecules, possibly due to the loss of exposed surface area and total hydration volume. Considering that patient’s blood contains other proteins in practical applications, experiments of mixture containing BSA or fetal bovine serum (FBS) were also been carried, including four mixtures: the antibody and gp120 mixture; antibody and BSA mixture; antibody, gp120 and BSA mixture; and antibody, gp120 and FBS mixture. They proved that BSA did not bind to the antibody, thus the binding signal of gp120 and antibodies can be separated from the background. This study proves that nanopores, as a label-free technology, provide a new way for drug design by establishing unique characteristic binding signals of antibody and antigens.

**Figure 4:**
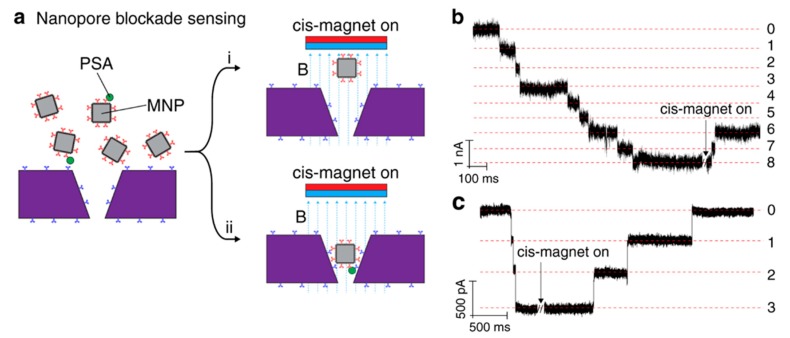
(**a**) Sketch map of active sensing of PSA by adjusting magnetic field. Event statistics of antibody and antibody + gp120. (**b**) Current traces when there was 5 pg/mL PSA. (**c**) Current traces when there was no PSA present. Reproduced under the terms of the Creative Commons Attribution License (CC-BY) [60]. Copyright 2019, The Authors, published by Nature Publishing Group.

Chuah et al. introduced anti-PSA (prostate-specific antigen) antibodies on the surface of silicon nitride nanopores, combined with anti-PSA modified magnetic nanoparticles, forming an immuno-sandwich to capture the PSA molecules (Figure 4a) [69]. If the PSA molecule is captured by magnetic nanoparticles, it cannot be removed when the magnetic field is reversed (cis-magnet on), but the unbound PSA magnetic nanoparticles can be removed (Figure 4a), thereby avoiding false signals. When the nanopore was blocked, long-term large changes in current were observed (Figure 4b). They used whole blood as a sample to measure PSA concentration, and the result shows a correlation between the number of detected irreversible blockades and PSA concentration, almost consistent with the concentration obtained by a commercial enzyme-linked immunosorbent assay (ELISA) kit. This work provided an idea of active sensing mechanism. Instead of waiting for the molecules to reach near the pore through diffusion, actively capturing them may be a solution to eliminate false signals and reduce the detection limit while improving specificity.

As a single-molecule biosensor platform with its high sensitivity of biomolecule detection, solid-state nanopores can achieve detection of single protein or its complex by instantaneous current drop. Some details need to be improved [69,70,71]. The frequency of the translocation events is significantly lower than the actual number of sample molecules. Some solutions are needed to make the molecules more easily to be captured. Low bandwidth and nanopore spatial-temporal resolution make some proteins undetectable yet. Increasing the frequency of data acquisition will also introduce more electrical noise, thus it is necessary to reduce electrical noise in solid-state nanopore devices even at high-frequency range [72].

## 3. Detection of Conformational Changes

Another important branch of protein research is the conformation or the size of the protein that is closely related to its function. Information of proteins such as charge, size, shape, and even amino acid sequence are reflected in the signal when they translocate across nanopore.

### 3.1. Characterizing and Distinguishing Proteins and Protein Oligomers

**Figure 5:**
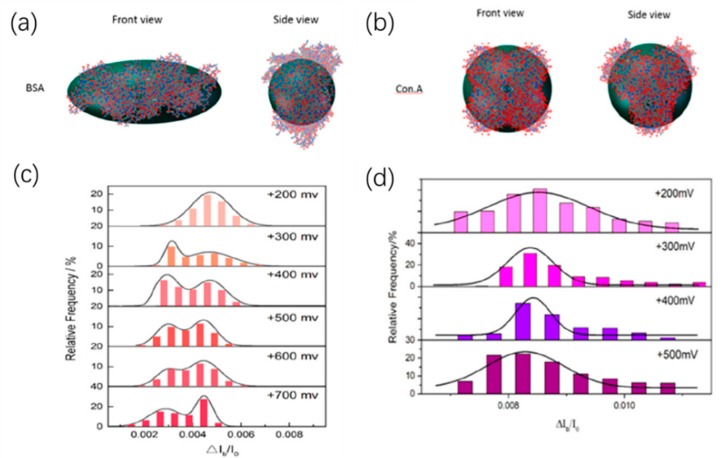
(**a**) Three-dimensional conformation of non-spherical BSA and (**b**) spherical con.A; and (**c**) histograms of the relative current blockade for non-spherical BSA and (**d**) spherical con.A. Adapted with permission from Anal. Chem. 2018, 90, 23, 13826–13831. Copyright 2018 American Chemical Society.

The amplitude of the current drop generated by the molecule through the nanopore is obviously related to the size of the molecule. The effect of protein size and structure on blocking signals was studied by Fologea et al. by comparing BSA and a larger protein fibrinogen [73]. On the contrary, the relative charge and size of protein molecules could be estimated based on the values of the mean blockage current ΔI_b_, the translocation duration t_d_, and the integrated area of a blockage A_ecd_. Likewise, they then demonstrated that nanopores could distinguish BSA of unfolded conformations and folded conformations [74]. They used denaturing agents guanidine hydrochloride, urea, and sodium dodecyl sulfate (SDS) plus dithiothreitol (DTT) to unfold the native state protein under different temperature. By measuring the relative current drop amplitude ΔI_b_/I_0_, solid-state nanopore devices can characterize the unfolding process of proteins. By computer simulations, they also showed that, in the nanopore translocation experiment, highly charged native state BSA molecules might undergo partial denaturation under the effect of voltage when entering the nanopore, which was consistent with previous reports [75]. Recently, Sha et al. observed the dynamics of the spherical and non-spherical proteins inside the solid-state nanopore under the electric field by the solid-state nanopore [76]. The prolate shape of BSA and the spherical shape of con.A could be distinguished by the relative current blockade (ΔI_b_/I_0_) (Figure 5). Interestingly, at low voltage, only one blockade current level was observed, while at high voltage larger than 300 mV, non-spherical protein BSA showed two obvious blockade current levels. By performing all-atom molecular dynamics simulations, it was found that, when the voltage is greater than a certain threshold value, the non-spherical protein BSA will show two preferential orientations instead of protein unfolding, resulting in two levels of blocking current. However, for spherical proteins, its orientation did not change the mean cross-sectional area; therefore, the ionic current could not be affected. Actually, in their experiments, there was only one blockade current level. This result demonstrates the ability of solid-state nanopores to characterize the behavior of proteins in solution, although there is still much room for improvement in detection accuracy to analyze more detailed translocation dynamics.

Utilizing the interaction of NTA–Ni^2+^ and His6-tag, Wei et al. reported that metallized silicon nitride nanopores chemically modified with nitrilotriacetic acid (NTA) receptors could be used for observing reversible binding and unbinding of the his-tagged proteins to the receptors [77]. The dissociation rates could be quantitively determined by analyzing the dwell time of binding events. Although the dwell time also depended on the binding location within the pore due to the uneven electric field strength inside the nanopore, measuring under series of different applied voltages could solve this problem. Protein and receptor’s binding time decreased with increasing voltage at any location. Therefore, the dissociation rates could be obtained through voltage-dependence. Using this strategy, they discriminate between the subclasses of rodent immunoglobulin G (IgG) antibodies. This work provides an extensive method to detect a certain protein or its interaction with other molecules as His-tag is a widely used label. Moreover, it presents an idea for protein identification and quantification in mixed analytes combined with specific biochemical selectivity.

Bilayer-coated solid-state nanopores were proved to have the ability to determine the approximate shape, volume, charge, rotational diffusion coefficient and dipole moment of individual proteins [78]. The proteins were tethered to the lipid anchor to slow down their translocation. Two methods were used to estimate the shape and volume of the protein sample. The first idea was based on direct proportionality between ΔI (current drop) and γ (electrical shape factors), and the geometrical relationship between γ and m (length-to-diameter ratio) of a spheroid, which needed to collect many translocation events to obtain distributions of maximum ΔI values. The second strategy estimated the shape and volume of proteins by analyzing single resistive pulses from the beginning to the end of individual translocation events, which lasted at least 400 μs. Although the first one is more accurate for shape estimation, the second one is the first strategy to estimate the shape and volume of a single protein molecule in situ in real time, and can also determine the rotational diffusion coefficient and dipole moment of a protein. In their later work, they successfully determined the ellipsoidal shape, volume, and dipole moment of single protein, which was not tethered to the lipid anchor [79]. This allowed free diffusion of the protein and increased the number of events available for analysis. They analyzed resistive pulses of single event with a duration of at least 150 μs instead of 400 μs due to faster translocation of proteins. This progress presented a promising possibility that solid-state nanopores may be able to characterize more subtle differences of proteins, at a completely native state, without any labeling or modification, even though solid-state nanopores do not affect any properties of proteins.

**Figure 6:**
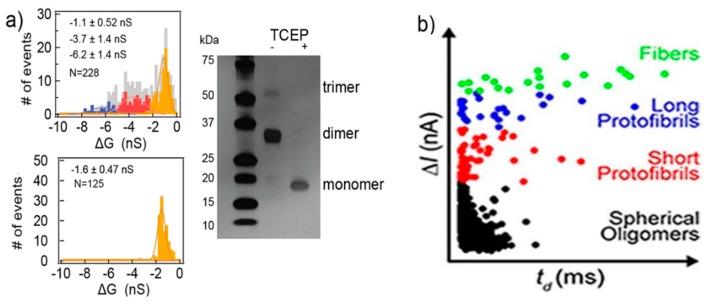
(**a**) The amplitude histogram of VEGF translocating events. SDS gel electrophoretic image of VEGF before and after reduction (right panel); and (**b**) Aβ conformation analysis using a lipid bilayer-coated nanopore based on ΔI of translocation signals. (**a**) Reproduced under the terms of the Creative Commons Attribution License (CC-BY) [80]. Copyright 2018, The Authors, published by Nature Publishing Group. (**b**) Reprinted with permission from ACS Nano 2012, 6, 7, 5909–5919. Copyright 2012 American Chemical Society.

Silicon nitride nanopores can characterize protein aggregates [80]. The protein they studied was vascular endothelial growth factor (VEGF), playing a pivotal role in stimulating vascularization, angiogenesis, and other normal or pathological physiological process. VEGF can form homodimers through disulfide bonds. Three populations of current blockades were observed in their experiments. Then, tris(2-carboxyethyl)phosphine (TCEP) was used to reduce the protein’s disulfide bonds. Only one population remained after the treatment, corresponding to the VEGF monomer, while the other two represent the VEGF the dimers and trimers, respectively (Figure 6a). They also researched the conformational change under different pH and concentration, as well as the effect of plasmin. Modifying and adjusting the surface of the pore can improve its functionality making it more flexible [81,82,83]. Yusko et al., inspired by the olfactory sensilla of insect antennae, used a fluid lipid bilayer coated nanopores, to fine-tune their pore size and also prevent pores from being blocked by proteins and eliminate non-specific binding [46]. Amyloid β (Aβ) oligomers of different sizes and shapes were easily discriminated through their translocation signals. Four kinds of populations were observed: spherical oligomers, protofibrils with lengths shorter than the length of the nanopore, protofibrils with lengths longer than the length of the nanopore, and mature fibers (Figure 6b) [84]. The sizes determined from resistive-pulse analysis were basically consistent with those analyzed by transmission electron microscopy. These studies show that solid-state nanopores can be developed as a real-time detection method to characterize native molecular structures and dynamics, where molecular immobilization and labeling become unnecessary.

### 3.2. Exploring Induced Protein Conformational Changes

**Figure 7:**
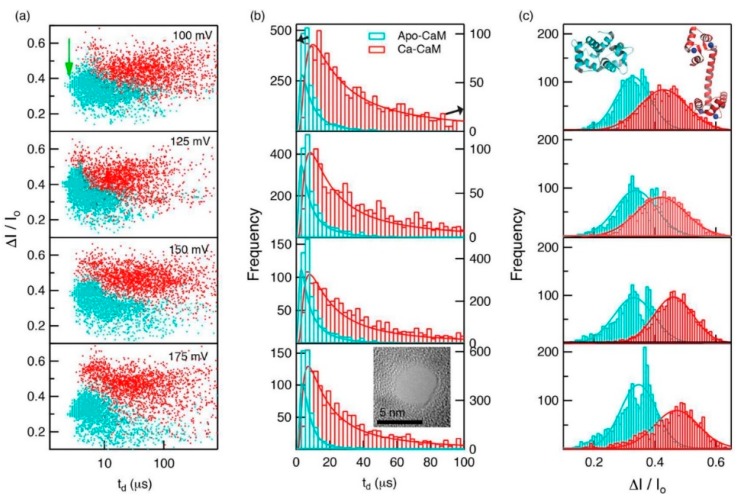
(**a**) Scatterplots of fractional current blockades vs. dwell times for Apo-CaM and Ca-CaM; (**b**) dwell-time distributions for Apo-CaM and CaCaM; and (**c**) fractional current amplitude distributions for Apo-CaM and CaCaM. Reprinted with permission from ACS Nano 2017, 11, 6, 5706–5716. Copyright 2017 American Chemical Society.

Proteins in organisms have high pH responsiveness. Their charge and structure are affected by pH and certain ions or ligands, thus performing corresponding biological functions through conformational changes. Waduge et al. first found a correlation between the shape of the current signal amplitude distribution and protein fluctuation by molecular dynamics simulations [85]. They also studied the conformational changes of calmodulin, from a calcium-free structure to a calcium-loaded structure using nanopores composed of both silicon nitride and HfO_2_. They added CaCl_2_ to the sample chamber after characterizing calcium-free calmodulin and saw significant changes in dwell time and fractional current blockade amplitude, which could be attributed to the conformational change of calmodulin (Figure 7).

**Figure 8:**
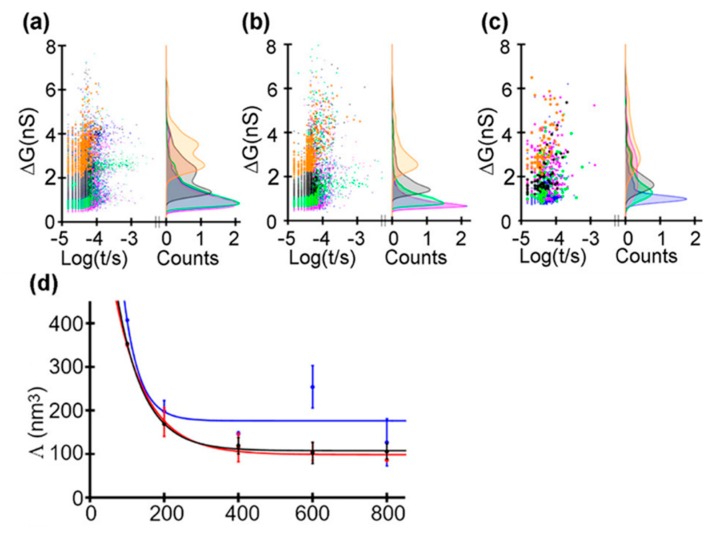
Translocating events of hSTf at: (**a**) pH 8; (**b**) pH 6; and (**c**) pH 4. Lines of different color represent different voltages: 800 mV (blue trace), 600 mV (magenta trace), 400 mV (green trace), 200 mV (gray trace), and 100 mV (orange trace). (**d**) Excluded volume with applied voltage at pH 8 (black trace), pH 6 (red trace), and pH 4 (blue trace). Solid lines correspond to an exponential fit. Adapted with permission from ACS Nano 2019, 13, 4, 4246–4254. Copyright 2019 American Chemical Society.

Recently, Saharia et al. investigated the voltage and pH responsiveness of hSTf protein using silicon nitride nanopores [59]. The hSTf is the main iron-containing protein in plasma. When a transferrin protein loaded with iron encounters a transferrin receptor on the surface of a cell, it binds to it and is transported into the cell in a vesicle by receptor-mediated endocytosis [86]. The hSTf has two globular lobes which contain two iron binding sites respectively. At pH higher than the protein’s pI, the hSTf tends to bind with Fe(III), forming a folded structure (holo form), while when pH value is under pI, the lobes open (apo form). In this way, they performed several translocation experiments at different pH and voltage (Figure 8). They calculated the transiently excluded volume of electrolyte and found that the excluded volume increased as the voltage decreased. This is understandable because when proteins are unfolded under the effect of high voltage, the internal voids will be exposed resulting in the decrease of molecular volume. Above 400 mV, the excluded volume remained relatively constant with applied voltage, which might represent the full extension of protein (Figure 8b). At pH 8, differences in the ΔG distribution of apo and holo forms could be observed. At 100 mV, the later had a larger conductance change due to its folded structure. However, as the voltage increased, the voltage induced unfolding exceed chelation effect of Fe(III). Then, the mixture of the two hSTf forms was added to the chamber, yielding similar results as experiments of individual holo-hSTf and pure apo product runs. The hSTf is responsible for the delivery of insoluble iron to cells and is very critical for iron homeostasis in humans. These nanopore-based methods mentioned above are potentially able to serve to detect an unusual presence of certain proteins, and investigate structural and dynamics properties of proteins, with great promise for clinical detection.

**Figure 9:**
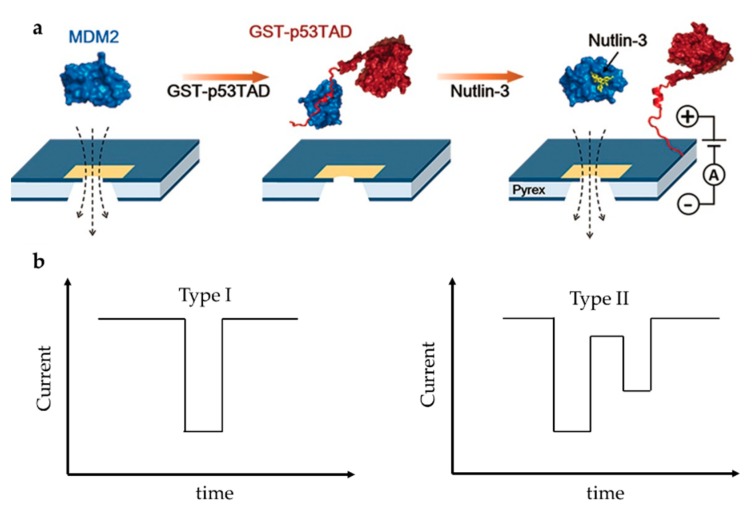
(**a**) Sketch map of MDM2/GST-p53TAD and its interaction with Nutlin-3; and (**b**) schematics of unimodal signal (left) and bimodal signal (right). (**a**) Adapted with permission from Angew. Chem.-Int. Edit. 2016, 55, 19, 5713–5717. Copyright 2016 WILEY-VCH Verlag GmbH & Co. KGaA, Weinheim.

On the other side, drug screening based on solid nanopore is a research hotspot but also challenging. Chae et al. designed a protein complex structure to examine the interaction of mouse double minute 2 protein (MDM2) and globular glutathione-S-transferase tagged p53TAD (GST-p53TAD) [87]. p53 is an important kind of tumor suppressor protein, which can be inhibited by MDM2 via a direct interaction with the p53 transactivation domain (p53TAD). A kind of MDM2 antagonist, Nutlin-3, can bind to MDM2 thus preventing the binding of p53TAD and MDM2, and restoring p53′s function to induce apoptosis of cancer cell. They added Nutlin-3 to the MDM2/GST-p53TAD complex and observed the translocation signal of free MDM2, which indicated that Nutlin-3 released MDM2 from the binding complex, thereby destroying MDM2/GST–p53TAD interaction (Figure 9a). The experiment was repeated with the negative control ABT-737, which is an inhibitor of Bcl-2 (B-cell lymphoma 2) family proteins and does not bind to MDM2. Unlike Nutlin-3, ABT-737 did not restore MDM2 translocation, confirming the specific inhibition of Nutlin-3. They then examined the conformational changes of MDM2-linker-p53TAD complex (MLP) induced by Nutlin-3 [88]. The linker’s function is to make MDM2 and p53 always be a whole. Nutlin-3 disrupted the interaction between p53TAD and MDM2 due to its relatively high affinity with MDM2, which made the MLP from near spherical form a dumbbell-like structure (Figure 9a). A shift in signal type was observed as expected at different Nutlin-3 concentrations. Spherical MLP produced a unimodal signal (Type I), while dumbbell-shaped MLP produced a bimodal signal (Type II) (Figure 9b). As the concentration of Nutlin-3 increased, the Type I signal decreased and the Type II signal increased. However, because all possible interactions between Nutlin-3 and MDM2 could not be guaranteed, the proportion of Type II signals was far lower than the theoretical value. In addition, due to the limitation of resolution, Type II signals might also be detected as Type I signals. Compared to fluorescence resonance energy transfer (FRET) and NMR, solid-state nanopore can be developed as a fast and label-free detection technique for efficient discovery of drug molecules based on protein–protein interactions.

In conclusion, the use of solid-state nanopores for protein characterization and detection of protein conformation has made some progress, providing a basis for disease research and drug screening. Nevertheless, the information obtained from the detected signals is limited, and many phenomena remain unable to be explained. To detect proteins more sensitively and accurately, it is necessary to slow the speed of protein translocation, and further enhance the spatial and temporal resolution by improving the nanopore devices. Furthermore, new preparation materials may need to be developed to achieve low noise; for example, two-dimensional (2D) materials such as graphene [89,90] and MoS_2_ [91,92] are effective for such problems.

## 4. Protein Sequencing using Solid-State Nanopores

Nanopore-based DNA sequencing has established a commercial platform, such as Oxford Nanopore Technologies, which demonstrated the ability of nanopores to discriminate DNA bases [17,21,93]. The development of single-molecule sequencing of proteins has lagged behind. In 1949, Edman et al. developed a method for determining the amino acid sequence one by one from the free N-terminus of a polypeptide chain [94]. The N-terminal amino acid residue is modified with phenyl isothiocyanate, and then cut from the polypeptide chain and identified by chromatography or electrophoresis. The remaining polypeptide chains are recycled to the next degradation and identification. However, since Edman degradation is based on the modification of the N-terminal amino group by chemical reagents, if the peptide is acetylated at the n-terminus or blocked by other chemical groups, sequencing cannot be performed directly. In addition, non-α-amino acid will stop sequencing. Mass spectrometry is currently the gold standard for protein sequencing [95,96]. Proteins are digested into peptides, the complex peptide mixture is then separated and analyzed using a tandem mass spectrometer, acquiring large numbers of tandem mass spectra. The tandem mass spectra are used to search a protein database to identify the proteins [95]. However, it cannot provide complete sequence information, and normally in complex biological samples, the interested protein content is quite low, and is difficult to amplify in vitro. These are the challenges of mass spectrometry methods [97,98]. A fast and high-throughput protein de novo sequencing technique on single-molecule level urgently needs to be developed.

Solid-state nanopores have shown their potential in DNA sequencing [91,99], although higher bandwidth and signal-to-noise ratio are required due to the extremely short dwell time of a single nucleotide [43,100]. It is expected that the solid-state nanopore sensor can be used to quickly detect the primary structure of a single protein, that is, the amino acid sequence. However, such similar high-throughput protein sequencing technology is not an easy task, and the following factors need to be considered. Firstly, compared to the relatively simple spatial structure of DNA, the natural folding structure of proteins makes nanopore-based protein sequencers require unfolding structure. Secondly, the proteins’ surface is not uniformly charged, and its translocation cannot be controlled simply by electrophoretic force, as discussed above [58]. Thirdly, proteins are difficult to amplify, which is a challenge for sensitivity. Finally, it is essential to extract and distinguish signals from twenty amino acids, while DNA has only four different standard bases.

To determine the sequence of amino acids one by one, the first step is to make the protein unfold linearly. Many researchers have made progress in promoting protein unfolding. Some physical conditions such as high temperature [75,101,102] and high voltage [75,103,104] can promote the unfolding of protein conformation. Freedman et al. detected thermal unfolding of BSA and various forms of BSA at high electric field [75]. They found that thermal denaturation leads to the loss of natural structures, but not necessarily fully unfolding, while the electric field strength might be sufficient to unfold the protein almost completely into an extended conformation. However, it was obvious that both treatments would lead to reduced translocation time. If the time resolution cannot be improved, some events would be undetected mistakenly, and the distinction between simple collision and translocation events would be more difficult.

**Figure 10:**
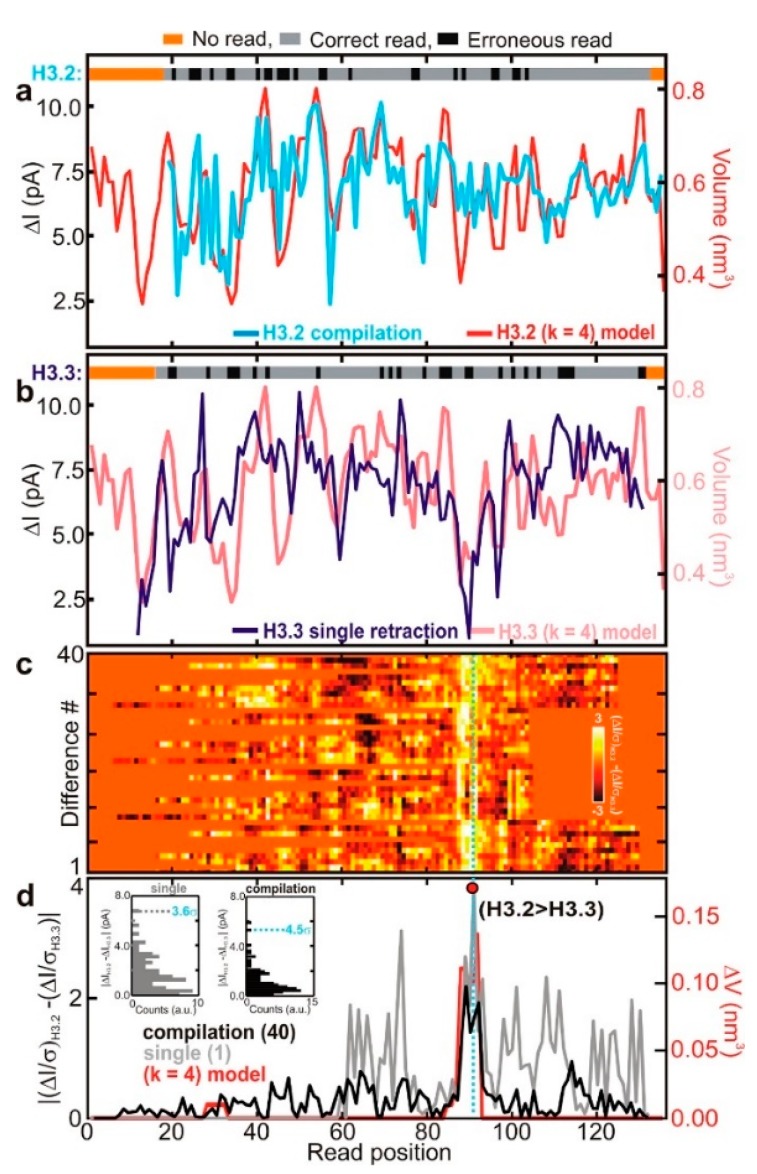
Sequence analysis of protein (**a**) H3.2 variant and (**b**) H3.3 variant; (**c**) heat map of differences of H3.2 and H3.3 current blockades; and (**d**) the red line shows the position of different amino acid between H3.2 and H3.3. Reprinted with permission from ACS Nano 2017, 11, 6, 5440–5452. Copyright 2017 American Chemical Society.

Another solution is using denaturants. SDS [105], guanidium chloride [106], urea [75], and other denaturants can destroy protein spatial structure to allow it to translocate through nanopores in the unfolded state. SDS denatures proteins and negatively electrifies linear peptide chains. Restrepo-Pérez et al. revealed that SDS could cause significant unfolding of proteins by molecular dynamics (MD) simulations combined with experiments [105]. As proteins have more negative electrical charge after being treated with SDS, their translocation is mainly determined by electrophoresis. In their experiments, a high concentration of SDS was found to make the electrophoretic force dominant, and the direction of protein transport was easier to define and control. Likewise, Kennedy et al. detected the proteins denatured by SDS and β-mercaptoethanol (BME) [107]. They used a solid-state nanopore with a diameter smaller than 0.5 nm and found that there was a correspondence between fluctuations and amino acid residues while the amplitude of the fluctuations was highly related to the volume blocked by four successive residues in the primary structure. Later, by utilizing SDS to stretch the proteins, two kinds of histones, which were different in only four residues, were detected using a sub-nanopore with a 0.5 nm diameter at the waist [108]. The current fluctuations showed a distinctly different peak near the replacement (Figure 10), which was related to the exclude volume [108].

The work of Kennedy and Dong et al. suggest the potential using nanopores to differentiate individual amino acid [107,108], although the exact type of amino acid could not be obtained. The lack of spatial resolution of solid-state nanopores precludes single amino acid identification. Individual protein translocates through the nanopore on time scales of 1 ms [109] making it difficult to characterize current blockade measurements at typical 100 kHz and lower measurement bandwidths, resulting in high event loss rate [54]. One method to solve the problem is to prolong the residence time of analytes. In the work of Ouldali et al., amino acids bound with short polycationic carriers are trapped inside the sensing region of the aerolysin nanopore, thus allowing sufficient time for sensitive measurement [110]. The “nanopore tweezer technology” is an alternative solution [111,112]. By engineering peptides flanked by oppositely charged tails at the N- and C- termini, during translocation, the ends of peptides experience oppositely oriented forces similar to an electrostatic “tug of war”. Thus, increasing the applied potential increases the polymer’s residence time inside the pore, regardless of the applied potential polarity [113,114].

**Figure 11:**
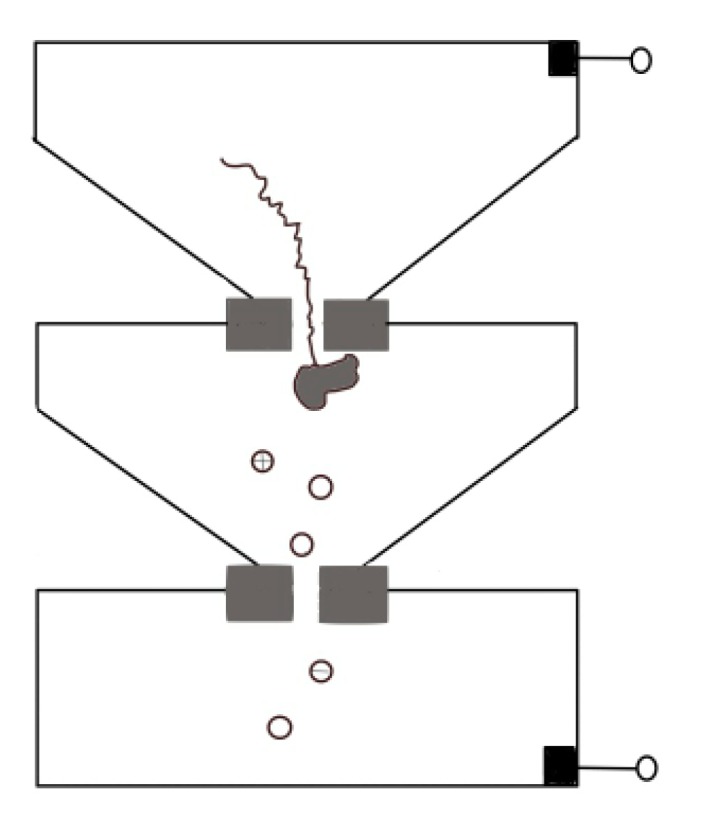
Sketch map of tandem nanopores for protein sequence.

Protein translocation speed can be slowed down by changing buffer conditions such as pH, or modifying the surface of pore to enhance the interaction between proteins and nanopore [46,77]. For both biological nanopores and solid-state nanopores [58,115,116,117], pH changes have a relevant effect on electroosmotic flow that refers to the net motion of the solvent induced by an applied voltage. The interplay between electrophoresis and electroosmosis can modulate the passage of a peptide. For a specific case of α-HL, low pH value increases its anion selectivity, leading to a corresponding increase in the electroosmotic speed of water against electrophoresis and thus reducing the net drift speed of the peptide [115,116,117]. Hu et al. provided a novel approach. They used pressure-driven flow to drive protein transport, and a reverse voltage was applied to retard transport [118]. Sampath put forward a novel idea [119]. Using two tandem nanopores, when the protein passes through the upstream nanopore, it is cleaved by exopeptidase, and the separated amino acid enters the downstream nanopore and extracts the signal there (Figure 11). Many obstacles need to be overcome in implementation, such as the rapid translocation through the upstream nanopore, which can be solved by a series of methods discussed above, and whether the statistical distribution of different amino acids’ signal through downstream can reach enough accuracy for distinguishing. On this point, Boynton et al. simulated the use of vertical nanochannels to stretch the protein in the vertical direction while recording the ion current in the horizontal direction [120]. They found that the distributions of ionic currents for each of the 20 amino acids were statistically different, which providing a possible approach for de novo protein sequencing.

**Figure 12:**
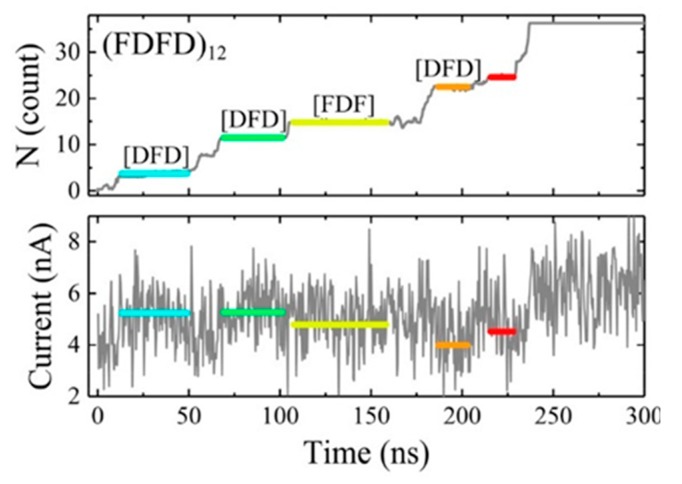
Translocation events of the (FDFD)_12_ peptide at a 600 mV. Adapted with permission from J. Phys. Chem. C 2018, 122, 4, 2070–2080. Copyright 2018 American Chemical Society.

On the other hand, because the thickness of the traditional solid film is too large to detect a single amino acid with a size of only 1 nm, an atomically thick carbon layer of graphene is theoretically thin enough to reach this goal. An all-atom molecular dynamics simulation was performed to prove this idea by Wilson et al. [90]. The peptides adhered to and moved along the surface of the graphene membrane, resulting in a slow and stepwise motion unidirectionally through graphene. The pores with 2.2 nm diameter produced a stepwise adjustment of ion currents related to the type of amino acids in the nanopore, and different ion current levels corresponded to different amino acid fragments. They suggested that the number of amino acid residues within the pore should be at a low level so that the types of amino acids were likely to be identified. Likewise, all-atom molecular dynamics simulation was also performed on MoS_2_ to research the peptides translocation and obtained similar results (Figure 12) [92]. Such 2D materials suggested possibilities to sequencing proteins because they are as thin as a single amino acid, thereby having high spatial resolution.

In summary, traditional protein sequencing methods such as Edman degradation, mass spectrometry, or a combination of the two have been applied to protein sequencing. However, the reads length of these methods is short, and they are often limited by the concentration and the structural properties of the sample. Single-molecule protein sequencing using solid-state nanopore technology can complement de novo sequencing of proteins without labeling, improve the sensitivity of existing technologies Despite the recent progresses, the main challenges of solid-state nanopore protein sequencing are spatial resolution (both lateral and vertical) and temporal resolution [121]. Thinner membranes and smaller holes are required, which represent fabrication challenges due to poor reproducibility, or researchers should strive for controlling analyte translocation. In addition, solid-state nanopores have higher noise level and lack of true atomic control. In the future, the technological innovations and novel device designs are expected to help us find more reliable solutions.

## 5. Conclusions

This article briefly introduces the basic principles and history of solid-state nanopores and reviews their development and applications in the fields of protein, protein and other molecule interactions, and conformational changes, as well as their potential for single molecule sequencing of proteins. For better developments and breakthrough, solid-state nanopore have to improve their resolution, both temporal and spatial. The temporal resolution issue is caused by excessively fast translocation of the biomolecules across the nanopore. Apparently, slowing down analytes’ translocation speed ameliorates the situation to some extent. Another way is expanding data acquisition bandwidth [72], but this could collect more electrical noise. Thus, in essence, the electrical noise in the solid-state should be reduced. At high bandwidths, amplifier noise becomes significant. Complementary metal-oxide semiconductors (CMOS) helps reduce the amplifier input capacitance [43,72]. Atomic layer deposition of Al_2_O_3_ is reported to be effective in fine-tuning the pore’s surface properties and reducing 1/f noise [35], which is attributable to the surface state of the inner nanopore wall. Lowering the chip capacitance is another crucial issue to reduce the total electrical noise [122,123]. A Si_3_N_4_ membrane coated with polyimide [122] and depositing a thick dielectric layer such as SiO_2_ underneath the pore membrane [36] reduces the membrane capacitance. Using SiO_2_ or polydimethylsiloxane (PDMS) as a replacement of the Si substrate in nanopore devices reduces the chip capacitance and the noise level [124,125].

The spatial resolution is related to pore size and thickness. Besides various 2D materials such as graphene, MoS_2_, and WS_2_, efforts of thinning of the SiN film have been reported in recent years. Thinning the membrane is important for high vertical resolution, but an ultrathin membrane has the risk of fracture. Yanagi et al. developed a new fabrication process that employs a polycrystalline-Si (poly-Si) sacrificial layer providing mechanical robustness to the membrane [126]; the effective thickness of the nanopores was estimated to range from 0.6 to 2.2 nm with diameter smaller than 2 nm [127]. A SiN nanopore with a 0.5-nm diameter at the waist was sputtered using a tightly focused, high-energy electron beam in a scanning transmission electron microscope (STEM) [107,108,128]. On single-layer MoS_2_, electrochemical reaction successively removes individual atoms or unit cells to form a nanopore smaller than 1 nm in size [129]. Despite these developments of nanopore fabrication technique, in consideration of productization and commercialization, solid-state nanopores should be mass produced with high reproductivity. Uniform dimensions, structure, and device characteristics are required. In summary, solid-state nanopore is a promising platform for biosensing. With the in-depth research of nanopore technology and the combination with various technologies, it will promote the identification of low-abundance proteins and the realization of high-throughput protein sequencing, bringing revolutionary changes to proteomics research.

## Figures and Tables

**Table 1:** Zeta potential (ζ_protein_) and effective diameter (D_protein_) of holo-hSTf, and the event frequency at 800 mV (ef_800mV_) and −800 mV (ef_–800mV_) of holo-hSTf translocation for different pH. Reprinted with permission from ACS Nano 2019, 13, 4, 4246–4254. Copyright 2019 American Chemical Society.

	pH 2	pH 4	pH 6	pH 8	pH 10
**ζ_protein_ (mV)**	12.6 ± 1.0	9.2 ± 0.5	–4.9 ± 0.7	–7.7 ± 0.5	–10.3 ± 0.5
**D_protein_ (nm)**	10.9 ± 0.2	9.3 ± 0.1	7.9 ± 0.4	7.2 ± 0.8	8.3 ± 0.5
**ef_800mV_ (s^–1^)**	0	∼2	∼24	∼36	negligible
**ef_800mV_ (s^–1^)**	negligible	∼0.12	0	0	0

## Abbreviations

DNA	deoxyribonucleic acid
NMR	nuclear magnetic resonance
CD	circular dichroism
IR	infrared spectroscopy
AFM	atomic force microscope
FIB	focused ion beam
EBD	electron-beam drilling
CDB	controlled dielectric breakdown
PEG	polyethylene glycol
APTES	3-aminopropyltriethoxysilane
ALD	atomic layer deposition
CVD	chemical vapor deposition
BSA	bovine serum albumin
pI	isoelectric point
hSTf	human serum transferrin protein
HDL	high-density lipoprotein
LDL	low-density lipoprotein
dsDNA	double-strand DNA
RNAP	RNA polymerase
FBS	fetal bovine serum
PSA	prostate-specific antigen
ELISA	enzyme-linked immunosorbent assay
SDS	sodium dodecyl sulfate
DTT	dithiothreitol
NTA	nitrilotriacetic acid
IgG	immunoglobulin G
VEGF	vascular endothelial growth factor
TCEP	tris(2-carboxyethyl)phosphine
Aβ	amyloid β-protein
MDM2	mouse double minute 2 protein
GST-p53TAD	glutathione-S-transferase tagged p53TAD
p53TAD	p53 transactivation domain
Bcl-2	B-cell lymphoma 2
MLP	MDM2-linker-p53TAD complex
FRET	fluorescence resonance energy transfer
2D	two-dimensional
MD	molecular dynamics
BME	β-mercaptoethanol
CMOS	complementary metal-oxide semiconductors
PDMS	polydimethylsiloxane
STEM	scanning transmission electron microscope
